# First-principles study of phase transition, elastic and thermodynamic properties of ZnSe at high pressure

**DOI:** 10.1038/s41598-020-59687-9

**Published:** 2020-02-24

**Authors:** Tao Yang, Xuejun Zhu, Junyi Ji, Jun Wang

**Affiliations:** 1 School of Biological and Chemical Engineering, Panzhihua University, Panzhihua, Sichuan 617000 China; 2 College of Chemical Engineering, Sichuan University, Chengdu, Sichuan 610065 China

**Keywords:** Phase transitions and critical phenomena, Semiconductors, Structure of solids and liquids

## Abstract

The structural and elastic properties of ZnSe with B3 and B1 phases under different pressure have been investigated by the first principle method based on density functional theory. The obtained structural parameters of ZnSe in both B3 and B1 structures are in good agreement with the available values. The transition pressure of ZnSe from B3 to B1 was predicted as 14.85 GPa by using the enthalpy–pressure data, which is well in line with experimental result. According to the obtained elastic constants, the elastic properties such as bulk modulus, shear modulus, Young’s modulus, ductile/brittle behavior and elastic anisotropy as a function of pressure for polycrystalline of ZnSe are discussed in details. In the frame work of quasi-harmonic Debye model, the temperature and pressure dependencies of the Debye temperature and heat capacity of ZnSe are obtained and discussed in the wide ranges.

## Introduction

The II–VI semiconductor compounds have attracted extensively attention due to their remarkable physical properties^1–4^ and their numerous potential applications^5,6^. Edwards *et al*.^7^ firstly studied the pressure-induced structural phase transformations of II–VI group compounds. Since then, theoretical and experimental studies on the structural and physical behavior of II–VI materials under high pressure are investigated by several research groups.

ZnSe, a significant member of the II–VI semiconductor compounds, which is a light yellow solid compound with a band gap of 2.70 eV^8^. It is an excellent candidate for fabrication of visual displays and photodetectors, etc^9–12^. It is an essential prerequisite for material’s synthesis and application to investigate the fundamental physical properties of ZnSe. Therefore, a great deal of researches have been done on the optical^13,14^, structural^2,15^, electronic^16,17^, and thermodynamic^18–20^ properties of ZnSe at ambient pressure. According to the result of Edwards *et al*.^7^, there may be a phase transition for ZnSe with applied pressure. So several research groups studied on the high-pressure behavior of ZnSe and confirmed that it has some polymorphic structures with the pressure increase. Karzel *et al*.^21^ reported the polycrystalline ZnSe from the B3 phase to the B1 phase happens at 13.0 GPa which encouraged great interest for theorists studying of structural stability under elevated pressure. Smelyansky *et al*.^22^ reported the phase transition pressure data as 15 GPa from full-potential linear augmented plane wave (FP-LAPW). While Biering *et al*.^23^ calculated it as 12.94 GPa using the projector augmented wave (PAW) method. When the phase transition happens with the increasing pressure, the nature of crystal structure would be different. However, it is difficult to obtain the exact value of these properties under high pressure in experimental studies. But the fundamental physical properties at elevated pressure, are extraordinary significances for the condensed matter physics, which will contribute to the understanding of the nature of materials. Therefore, the theoretical study could be a powerful tool to acquaint the ZnSe under elevated pressure, owing to the advance in theoretical methods. However, to the best of my knowledge, there are only a few references investigating the elastic and thermodynamic properties of the B3-type ZnSe. Especially, the behaviors of elastic and thermodynamic properties of the B1-type ZnSe are rarely considered under high pressure. In this work, we have focused on the structural phase transition and the elastic properties as a function of pressure for both B3 and B1 phases by using plane-wave pseudopotential density functional theory (DFT). Meanwhile, some detailed thermodynamic property at elevated pressure and temperature have been calculated through the quasi-harmonic Debye model^24^.

## Theoretical Methods

The calculations in present work are performed using the pseudopotentials plane-wave approach in the frame of density functional theory (DFT) as implemented in Cambridge Serial Total Energy Package (CASTEP) code^25,26^. For structural calculations, the Perdew-wang-1991(PW91)^27^ formulation of the generalized gradient approximation (GGA) was chosen as the optimum exchange correlation of electrons. The Broyden–Fletcher–Goldfarb–Shannon (BFGS) algorithm^28^, which provides a very efficient method to achieve the geometry with a minimum energy, was applied to relax the crystal structure to reach the ground state. In order to obtain an optimum geometric, the kinetic energy cutoff is set as 500 eV and *k* point separation in the Brillouin zone of the reciprocal space is 8 × 8 × 8 for both B3 and B1 phases. Pseudo atomic calculations of ZnSe are performed for Zn 3*d*^10^4*s*^2^ and Se 4*s*^2^4*p*^4^. The space group of B3 structure is *F43m*, the positions of the atoms Zn and Se are (0, 0, 0) and (0.25, 0.25, 0.25). The space group of B1 structure is *Fm3m*, the Zn and Se atoms are located at (0, 0, 0) and (0.5, 0.5, 0.5), respectively. To achieve some reliable results, the self-consistent convergence of the total energy is less than 5.0×10^−7^ eV/atom.

## Results and Discussion

### Pressure-induced structural phase transition

**Figure 1:**
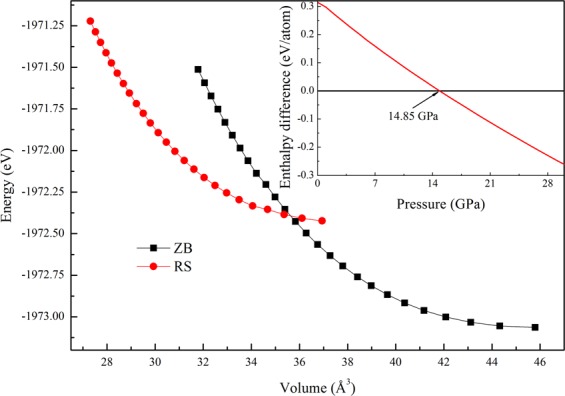
Total energy versus unit cell volume for the B3 and B1 phases of ZnSe. Inset: enthalpy difference as a function of pressure for ZnSe at *T* = 0 K.

For both B3 and B1 structures of ZnSe, a series of different values of primitive cell volume are set to calculate the total energy. The calculated total energies as a function of volume for both structures of ZnSe are displayed in Fig. 1. According to the result shown in Fig. 1, it is clear to see that the ZnSe with B3 structure is a more stable phase. In order to obtain the equilibrium lattice constants *a*, the bulk modulus *B*_0_ and its pressure derivative *B*^’^_0_, the total energy *E vs*. volume is fitted to the Birch-Murnaghan equation of states (EOS)^29^. The results are listed in Table 1, which are also compared with some other theoretical and experimental results. The calculated values of lattice parameters are slight overestimated and the bulk modulus are little underestimated corresponding to the experimental data^21^. The overestimation in the lattice parameters and underestimation in the bulk modulus is a common feature with GGA^30,31^. However, the calculated values using GGA for both B3 and B1 phases agree well with the corresponding experimental value^21^ and some available theoretical data^2,22,23,32,33^. In order to find out the most stable phase at finite pressure, the free energy of two structures under different pressures should be calculated. According to thermodynamic stability theory, the lower phase free energy corresponds to the more stable phase. It is well known that the free energy defined as *G* = *E* + *PV-TS*. The last term (*TS*) can be neglected because our density functional calculations are essentially performed at 0 K. So the free energy *G* reduces to enthalpy relation *H* (*H* = *E* + *PV*). When the phase transition happens, the enthalpies of two different structures would be the same. The pressure dependence of the two phases ZnSe are illustrated in the inset of Fig. 1. It is obvious from the inset of Fig. 1 that the enthalpy curve of the B3 structure crosses that of the B1 structure around 14.85 GPa, implying that a solid phase transition from B3 to B1 induced by pressure occurs at 14.85 GPa. The obtained phase transition pressure is quite agree with the previous experimental result^21^ and some other theoretical calculations^22,23^.

**Table 1:** The obtained lattice constant (*a*), bulk modulus *B*_0_, and the pressure derivative of bulk modulus (*B*^’^_0_) of ZnSe in both phases at *T* = 0 K, compared with the experimental result and some theoretical values.

Structure	Parameter	Present	Exp.	Theor.
B3	*a*	5.679	5.66^21^	5.66^2^, 5.75^32^, 5.63^33^, 5.66^22^, 5.82^22^
	*B*_0_	66.75	69.3^21^	63.0^2^, 57.3^32^, 72.4^33^, 62.4^22^, 52.9^22^
	*B*^’^_0_	3.94	4^21^	3.87^2^, 4.56^32^, 4.76^33^, 4.05^22^, 3.81^22^
B1	*a*	5.329	5.29^21^	5.31^2^, 5.38^32^, 5.28^22^, 5.42^22^, 5.37^23^
	*B*_0_	86.84	104^21^	77.8^2^, 75.5^32^, 92.1^22^, 74.1^22^, 71.7^23^
	*B*^’^_0_	3.92	4^21^	3.75^2^, 3.6^32^, 3.47^22^, 3.55^22^, 4.7^23^

### Single-crystal elastic constants and related properties

As is known to all, the elastic constant is very important parameter for the elastic material and can represent the amount of the elasticity of a material. To investigate the elastic constants of ZnSe, the non-volume conserving method is applied. The elastic constants *C*_*ijkl*_ can be described as follows^34,35^

1 $${C}_{ijkl}={\frac{\partial {\sigma }_{ij}}{\partial {e}_{kl}}|}_{X}={\frac{1}{V}\frac{{\partial }^{2}E(x)}{\partial {e}_{ij}\partial {e}_{kl}}|}_{X}$$

**Figure 2:**
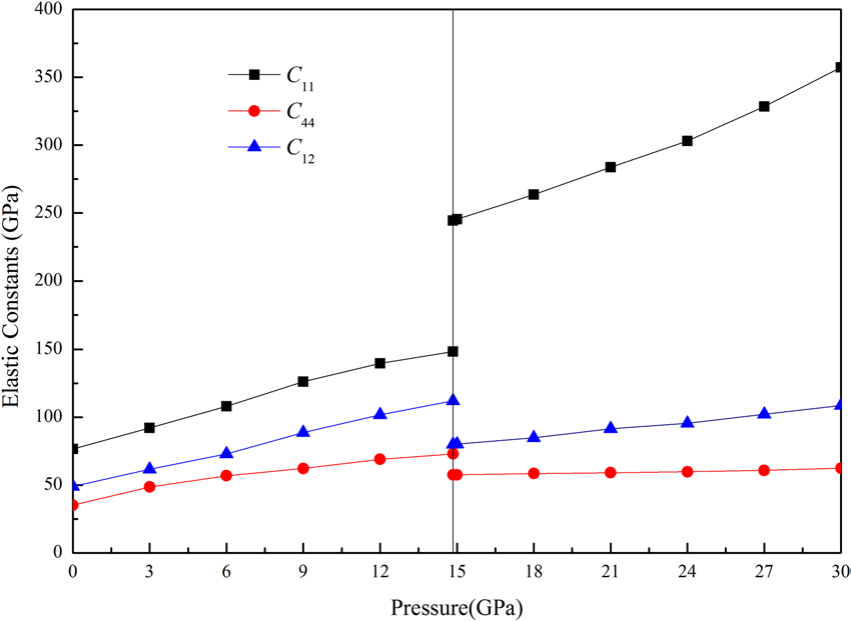
Elastic constants as a function of pressure for both B3 and B1 phases of ZnSe.

Where *σ*_ij_ stands for the applied stress, *e*_kl_ corresponds to the Eulerian strain tensors, *X* denotes the coordinate. The fourth-rank tensor *C* has generally 21 independent components. If the symmetry of the system is account into consideration, the number of independent components reduces. For a cubic structure, there are only three independent elastic constants, namely *C*_11_, *C*_12_, and *C*_44_. The obtained elastic constants for both phases have been given in Table 2 which also contains some experimental and theoretical values for comparison. The discrepancy between our computed results and the experimental values^36^ for the elastic constants of B3 structure is acceptable. Meanwhile there is well in line between our obtained results and some previous theoretical results^32,33,37,38^ for B3 phase. To the best of my knowledge, there are no experimental values of the elastic constants for B1 structure at elevated pressure. The calculated values in this work are not comparable with the work of Ji *et al*.^39^ by calculation. The variations of elastic constants as a function of pressure for both structures are shown in Fig. 2. It is observed that all the elastic constants for both structures in the considered range of pressure increase with increasing pressure showing a monotonic behavior. For both B3 and B1 phases, it is obvious that *C*_11_ vary more under the impact of pressure than others. The elastic constant *C*_11_ is related to the elasticity in length, which is changed with the longitudinal strain, and the *C*_12_ and *C*_44_ are represented the elasticity in shape. Therefore, the pressure has a much more significant influence on elasticity in length. Moreover, the behavior of elastic constants for B3 phase is still in line with the work of Wang *et al*.^37^ by calculation.

**Table 2:** The obtained elastic constants of ZnSe compared with other results.

Structure	Reference	*C*_11_	*C*_12_	*C*_44_
B3	Present work	73.43	45.79	34.83
	Theor.	91^33^	63^33^	59^33^
		84.0^32^	49.0^32^	55.8^32^
		91.2^37^	58.2^37^	42^37^
		82.45^38^	42.71^38^	35.5^38^
	Exp.	81^36^	48.8^36^	44.1^36^
B1	Present work	244.37	79.90	57.52
	Theor.	154.852^39^	112.7^39^	41.571^39^

### Polycrystalline elastic moduli and related properties

For the sake of ensuring the mechanical stability of ZnSe, the elastic constants of ZnSe in the considered range of pressure should satisfy the mechanical stability criteria. The bulk modulus *B* and shear modulus *G* are parameter that must be investigated. The reason is that the bulk modulus *B* is the macroscopic property of a material which reflects the material’s resistance to homogeneous compression and the shear modulus *G* represents the resistance of the material to shear strain. In order to obtain the bulk modulus *B* and shear modulus *G*, the Hill model is applied, which takes the arithmetic average of the Voigt^40^ and Reuss models^41^. For cubic system, Hill shear modulus *G* and bulk modulus *B* are taken in the form of^42^:

2 $$B=\frac{1}{2}({B}_{R}+{B}_{V}),G=\frac{1}{2}({G}_{R}+{G}_{V})$$

where *B*_*R*_ denotes Reuss bulk modulus, *B*_*V*_ represents Voigt bulk modulus, *G*_*R*_ is Reuss shear modulus, *G*_*V*_ is the Voigt shear modulus, given as:

3 $${B}_{V}={B}_{R}=\frac{1}{3}({C}_{11}+2{C}_{12})$$

4 $${G}_{V}=\frac{1}{5}({C}_{11}-{C}_{12}+3{C}_{44})$$

5 $${G}_{R}=\frac{5({C}_{11}-{C}_{12}){C}_{44}}{4{C}_{44}+3({C}_{11}-{C}_{12})}$$

The Young’s modulus *E* can be regarded as an index to measure the difficulty of producing elastic deformation. It can be given by

6 $$E=\frac{9BG}{G+3B}$$

**Figure 3:**
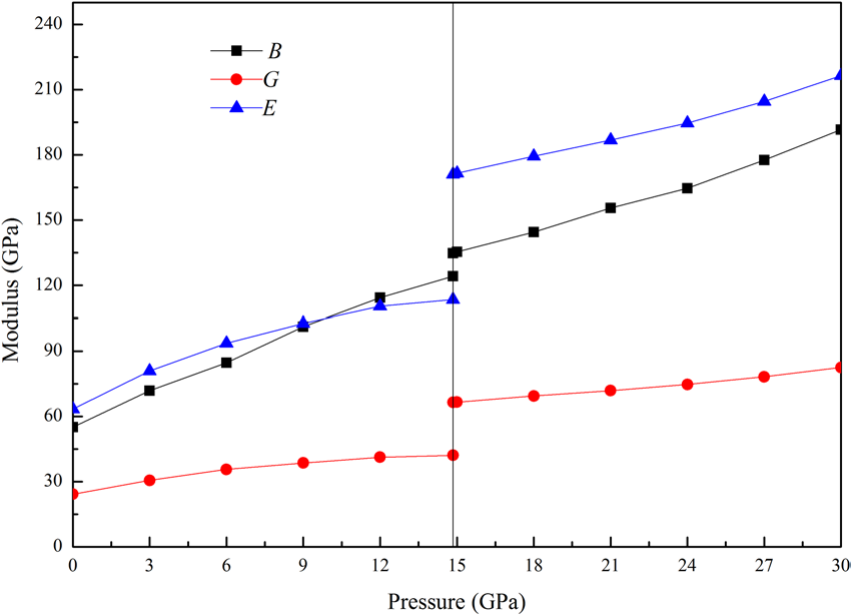
Bulk modulus *B*, shear modulus *G*, Young’s modulus *E* as a function of pressure for both B3 and B1 phases of ZnSe.

Figure 3 depicts the pressure dependence of the bulk modulus *B*, shear modulus *G*, and Young modulus *E* of ZnSe in both structures. It is obvious from Fig. 3 that all these elastic moduli (*B*, *G* and *E*) for both B3 and B1 phases increased monotonically with the increasing pressure in the considered range of pressure. The effects of the pressure on *B* and *E* are larger than that on *G*, indicating that the pressure can significantly improve the anti-compression ability and stiffness of ZnSe. The above results show that the increasing pressure can enhance the elastic properties of ZnSe.

**Figure 4:**
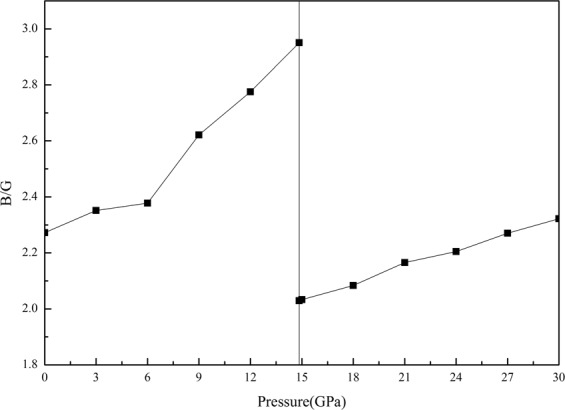
The B/G ratio as a function of pressure for both B3 and B1 phases of ZnSe.

In order to distinguish the brittle (ductile) behavior of materials, an important empirical relationship, which is the value of *B/G*, has been proposed by Pugh^43^. The critical threshold value for analyzing the brittle–ductile behavior of materials is approximately 1.75. As the ratio is up to 1.75, the material shows as a ductile manner. Otherwise, it would possess a brittle character. Figure 4 describes the behavior of *B/G* ratio for both B3 and B1 structures in the investigated pressure range. The obtained *B/G* ratio for polycrystalline ZnSe are all larger than 1.75, implying that the polycrystal tend to perform in a ductile manner. Figure 4 further illustrates that the increase of hydrostatic pressure tends to enhance the ductile behavior of ZnSe. The effect of pressure on the ductile of B3 ZnSe is larger than that on the ductile of B1 ZnSe.

### Elastic anisotropy

**Figure 5:**
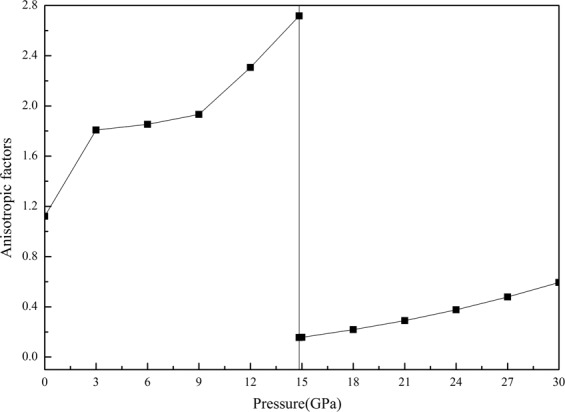
Universal anisotropy index as a function of pressure for both B3 and B1 phases of ZnSe.

The elasticity anisotropy of the material can reflect the difference properties between in two directions perpendicular. So it is necessary for crystal physics and engineering science to investigate the elasticity anisotropy of the material. The elastic anisotropy can be described the universal elastic anisotropy index *A*^*U*^ which is developed by Ostoja–Starzewski^44^ for crystal with any symmetry. The *A*^*U*^ can be written as follows:

7 $${A}^{U}=5\frac{{G}_{V}}{{G}_{R}}+\frac{{B}_{V}}{{B}_{R}}-6$$

where *B*_*R*_ denotes Reuss bulk modulus, *B*_*V*_ represents Voigt bulk modulus, *G*_*R*_ corresponds to Reuss shear modulus, *G*_*V*_ is the Voigt shear modulus, respectively. For the case of isotropic crystals, the universal elastic anisotropy index is equal to zero. Contrarily, any value deviate from zero implies the degree of single crystal anisotropy. Figure 5 shows the variation of the *A*^*U*^ obtained from our studies under surveyed pressure range. It is obvious that the universal elastic anisotropy index *A*^*U*^ for both phases are larger than zero and significantly increases with increase of pressure, indicating that the elastic anisotropy in both structures would rise rapidly in the investigated pressure range. The universal elastic anisotropy index of ZnSe in B1 phase increases in smaller slope, indicating the impact of pressure on B1 phase is smaller than that on B3 phase. The minimum value of *A*^*U*^ in B3 structure is still bigger than the maximum value of *A*^*U*^ in B1 structure in the pressure range of 0–30 GPa, revealing that ZnSe in B3 phase is more anisotropy.

**Figure 6:**
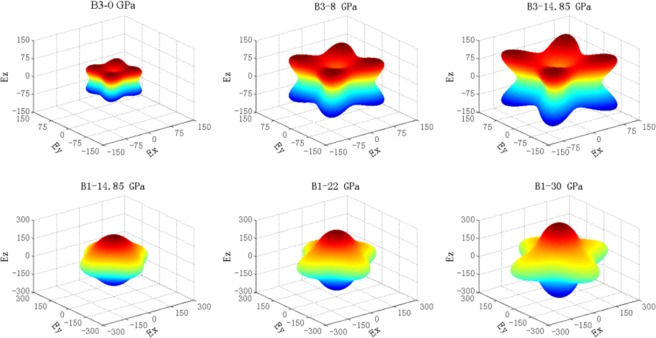
The surface construction of the Young’s modulus for both B3 and B1 phases of ZnSe (unit: GPa).

To investigate the anisotropic characteristics of ZnSe, a curved surface of a three-dimensional (3D) representation of the elastic anisotropy of the two cubic ZnSe single crystal was further attempted, which can be expressed by the directional dependences of reciprocals of Young’s modulus. For a cubic crystal system, the directional dependence of the Young’s modulus can be expressed as^45^.

8 $$\frac{1}{E}={l}_{1}^{4}{S}_{11}+{l}_{2}^{4}{S}_{22}+{l}_{3}^{4}{S}_{33}+2{l}_{1}^{2}{l}_{2}^{2}{S}_{12}+2{l}_{1}^{2}{l}_{3}^{2}{S}_{13}+2{l}_{3}^{2}{l}_{3}^{2}{S}_{23}+{l}_{1}^{2}{l}_{2}^{2}{S}_{66}+{l}_{1}^{2}{l}_{3}^{2}{S}_{55}+{l}_{2}^{2}{l}_{3}^{2}{S}_{44}$$

where *S*_ij_ corresponds to the elastic compliance constants and *l*_1_, *l*_2_ and *l*_3_ denote the direction cosines. Figure 6 displays the directional Young’s modulus of both phases under different hydrostatic pressures. The surface contours of the Young’s modulus of both structures become more anisotropic geometry with an increasing pressure, revealing that ZnSe in both B3 and B1 structures tend to become more anisotropic as the hydrostatic pressure increases. The obtained result is in good agreement with the response of the universal elastic anisotropy index *A*^*U*^ illustrated in Fig. 5. Figure 6 further depicts that the higher the pressure is, the larger the 3D figures of Young’s modulus is. This result agrees well with the trend of *E* depicted in Fig. 3. As for both structures at 14.85 GPa, the difference in their the 3D directional dependence of Young’s modulus is highly apparent. The 3D directional dependence of the Young’s modulus of B3 phase show remarkable anisotropic geometry, indicating that B3 ZnSe is more elastically anisotropic than B1 ZnSe. This result is also in agreement with the above results from the pressure-dependent the universal elastic anisotropy index *A*^*U*^ shown in Fig. 5.

### Thermodynamic properties

The Debye temperature not only reflects the degree of dynamic distortion of the crystal lattice, but also represents the interatomic binding force of the substance. Many physical quantities of the material are related to it, such as elasticity, hardness, melting point and specific heat. In order to investigate the thermodynamic properties of both phases of ZnSe, the Debye temperature versus temperature and hydrostatic pressure of both phases of ZnSe with a periodic boundary condition have been calculated. Meanwhile, the heat capacity as one of the most important thermodynamic properties was also given in this article. To explore the thermodynamic properties of both phases of ZnSe, the quasi-harmonic Debye model^24,46^ is carried out. In which the non-equilibrium Gibbs function G* (*V*; *P*, *T*) is defined the following form:

9 $${{G}}^{\ast }({V};{P},{T}){=}{E}({V}){+}{PV}{+}{{A}}_{{vib}}[{\theta }({V});{T}]$$

Where *E*(*V*) denotes the total energy per unit cell, *V* corresponds to the volume of the molecular system, *T* stands for the temperature of system, *P* represents the constant pressure condition, *A*_*vib*_ is the vibrational term, which can be written as^47,48^

10 $${A}_{vib}({\theta }(V);T)=nKT(\frac{9{\theta }}{8T}+3\,\mathrm{ln}(1-{e}^{-{\theta }/T})-D(\frac{{\theta }}{T}))$$

Where *n* is the number of atoms in the molecule, *D*(*θ/T*) indicates the Debye integral, and for an isotropic solid, *θ* is expressed as^49^

11 $${\theta }{=}\frac{h}{k}{[{6}{{\pi }}^{{2}}{{V}}^{{1}{/}{2}}{n}]}^{{1}{/}{3}}{f}({\sigma })\sqrt{\frac{{{B}}_{{s}}}{{M}}}$$

Where *M* is the molecular mass per formula unit, the *f*(σ) is described in detail^48,50^. *B*_s_ denotes the adiabatic bulk modulus, which is approximated written in the form of^46^

12 $${B}_{s}\cong B(V)=V\frac{{d}^{2}E(V)}{d{V}^{2}}$$

Therefore, the non-equilibrium Gibbs function G*(*V; P, T*) can be minimized with respect to volume *V* as follows:

13 $${[\frac{{\delta }{{G}}^{\ast }({V}{;}{P}{,}{T})}{dV}]}_{P,T}=0.$$

The thermal EOS *V*(*P, T*) can be obtained by solving Eq. (13). Furthermore, the heat capacity *C*_*v*_ is given by the following equation^51^.

14 $${C}_{v}=3nk[4D(\frac{{\theta }}{T})-\frac{3{\theta }/T}{{e}^{{\theta }/T}-1}]$$

**Figure 7:**
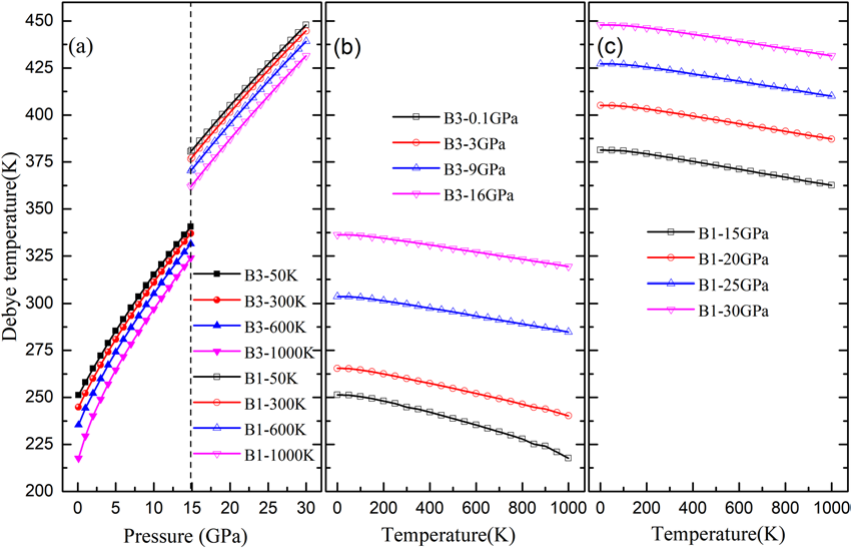
The Debye temperature of ZnSe as a function of temperature and pressure.

**Figure 8:**
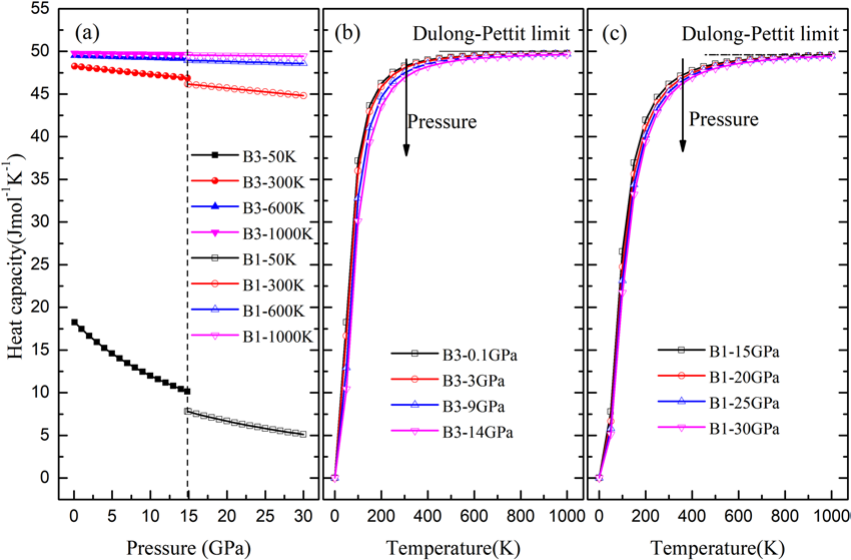
Heat capacity of ZnSe *vs*. temperature and pressure.

The Debye temperature *θ* and heat capacity *C*_*v*_ for both phases of ZnSe at different temperatures and pressures are displayed in Figs. 7 and 8, respectively. The pressure dependence of Debye temperature *θ* of ZnSe at different temperature is shown in Fig. 7(a). The obtained Debye temperature for B3 ZnSe at zero pressure and 300 K, is 244.42 K, which agrees well with the experimental result^19^. The value of Debye temperature for B1 ZnSe at 14.85 GPa and 300 K locates at 376.64 K. To the best of my knowledge, there is no comparable result of the Debye temperature for B1 ZnSe. So the calculated values will provide a basis for future research work. It is obvious from Fig. 7(a) that as the temperature is fixed, the Debye temperature of ZnSe increases almost linearly with an increasing pressure. It is also found the Debye temperature of B3 is smaller than that of B1 at 14.85 GPa. Figure 7(b,c) indicate that the trend of Debye temperature at a given pressure would decreases slightly with temperature. It is clear that the Debye temperature is sensitive to the pressure than the temperature.

As displayed in Fig. 8(a), the heat capacity *C*_*v*_ at a given temperature would decrease slightly with the increase of pressure. Under high temperature, the heat capacity is almost constant in the pressure range of 0–30 GPa. The decreasing rate of *C*_*v*_ is more obvious under low temperature (e.g. 50 K). The heat capacity for B3 phase at zero pressure and 300 K is 48.27 J·mol^−1^ K^−1^, which is reasonable compared with the available values^20^^.^ The *C*_*v*_ for B1 phase at 14.85 GPa and 300 K is 46.17 J·mol^−1^ K^−1^, which is reported for the first time, and provides a meaning reference for future research work. As displayed in Fig. 8(b,c), the heat capacity increase rapidly at temperature below the Debye temperatures and slowly approaches a linear increase and then the increasing rate of *C*_*v*_ is near zero. The acoustic vibrations play a dominant role in the vibrational excitations at temperatures below the Debye temperatures, so *C*_*v*_ strictly follows the T^3^-law^24^. The variation of the heat capacity *C*_*v*_ with temperature at intermediate temperatures is dominated by the details of vibration of the atoms. Because of the anharmonic approximation of the Debye model, *C*_*v*_ of ZnSe for a given pressure increases rapidly from 0 to 300 K. On account of the impact of anharmonic on the heat capacity *C*_*v*_ is suppressed at higher temperature, the heat capacity gradually approaches the Dulong–Pettit limit, which is common to all solids at temperatures far above the Debye temperature. It is remarkable that the temperature effect on *C*_*v*_ is much greater than the pressure effect on *C*_*v*_ of ZnSe.

## Summary and Conclusion

The structural and elastic properties of ZnSe for both B3 and B1 structures under different pressures are investigated *via* the first-principles plane-wave pseudopotential method based on density functional theory (DFT). From the usual condition of equal enthalpies, the phase transition of ZnSe from B3 to B1 occurs at the pressure of 14.85 GPa. According to the obtained elastic constants, the pressure dependence of the bulk modulus, shear modulus and Young’s modulus of ZnSe are calculated and discussed in detail. The increasing rate of *B/G vs*. pressure is remarkably, indicating that ZnSe in two phases is a ductile material and the ductility increases with pressure. The obtained anisotropic indexes and the direction dependence of the Young’s modulus demonstrate that ZnSe in B3 phase is more anisotropic than B1 phase and the elastic anisotropy of both phases become stronger with an increasing pressure. The thermodynamic properties of ZnSe, such as Debye temperature and heat capacity as a function of the pressure and temperature are successfully investigated by quasi-harmonic Debye modeling.

## Supplementary information

Supplementary Data.
