# Supplementary material for: First-principles study of phase transition, elastic and thermodynamic properties of ZnSe at high pressure
Source: Sci Rep. 2020 Feb 24;10:3265. doi: 10.1038/s41598-020-59687-9 (PMC7039993; doi:10.1038/s41598-020-59687-9)
Supplement: Supplementary file 1 — Supplementary Data. [file 41598_2020_59687_MOESM1_ESM.docx]

**First-principles study of** **phase transition, elastic and thermodynamic properties of ZnSe** **at high pressure**

Tao Yang^1^, Xuejun Zhu^1^, Junyi Ji^2^, Jun Wang^1*^

**Supplementary files**

| Structure | B3 | | | | Structure | B1 | | | |
| --- | --- | --- | --- | --- | --- | --- | --- | --- | --- |
| Pressure (GPa) | Debye temperature at a given temperature (K) | | | | Pressure (GPa) | Debye temperature at a given temperature (K) | | | |
|  | 50 K | 300 K | 600 K | 1000 K |  | 50 K | 300 K | 600 K | 1000 K |
| 0.1 | 251.23 | 244.79 | 235.33 | 217.64 | 14.85 | 380.53 | 376.64 | 370.34 | 361.82 |
| 1 | 258.06 | 252.25 | 244.24 | 229.43 | 15 | 381.26 | 377.39 | 371.1 | 362.6 |
| 2 | 265.3 | 259.99 | 252.12 | 240.22 | 16 | 386.15 | 382.34 | 376.11 | 367.69 |
| 3 | 272.24 | 267.29 | 259.9 | 248.99 | 17 | 390.97 | 387.21 | 381.04 | 372.69 |
| 4 | 278.91 | 274.24 | 267.2 | 257.11 | 18 | 395.72 | 392.01 | 385.89 | 377.6 |
| 5 | 285.37 | 280.9 | 274.13 | 264.61 | 19 | 400.39 | 396.73 | 390.69 | 382.44 |
| 6 | 291.62 | 287.32 | 280.77 | 271.66 | 20 | 405.02 | 401.39 | 395.4 | 387.2 |
| 7 | 297.7 | 293.53 | 287.16 | 278.36 | 21 | 409.57 | 405.99 | 400.04 | 391.89 |
| 8 | 303.61 | 299.56 | 293.33 | 284.78 | 22 | 414.04 | 410.52 | 404.62 | 396.52 |
| 9 | 309.37 | 305.42 | 299.32 | 290.99 | 23 | 418.47 | 414.99 | 409.13 | 401.09 |
| 10 | 314.99 | 311.12 | 305.14 | 296.98 | 24 | 422.83 | 419.42 | 413.58 | 405.59 |
| 11 | 320.49 | 316.69 | 310.82 | 302.8 | 25 | 427.15 | 423.75 | 417.99 | 410.04 |
| 12 | 325.86 | 322.13 | 316.36 | 308.47 | 26 | 431.4 | 428.07 | 422.33 | 414.44 |
| 13 | 331.11 | 327.45 | 321.77 | 314 | 27 | 435.61 | 432.3 | 426.62 | 418.78 |
| 14 | 336.25 | 332.66 | 327.06 | 319.4 | 28 | 439.76 | 436.49 | 430.86 | 423.05 |
| 14.85 | 340.56 | 337 | 331.47 | 323.89 | 29 | 443.86 | 440.64 | 435.05 | 427.29 |
| - | - | - | - | - | 30 | 447.91 | 444.73 | 439.19 | 431.47 |

**Supplementary table 1.** The Debye temperature of ZnSe under both phases as a function of pressure at a given temperature. These data obtain from Figure 7(a).

.

| Temperature (K) | Debye temperature at a given Pressure (GPa) | | | |
| --- | --- | --- | --- | --- |
|  | 0.1 GPa | 3 GPa | 9 GPa | 14 GPa |
| 0 | 251.39 | 265.43 | 303.69 | 336.31 |
| 50 | 251.23 | 265.3 | 303.61 | 336.25 |
| 100 | 250.44 | 264.62 | 303.12 | 335.87 |
| 150 | 249.32 | 263.66 | 302.32 | 335.19 |
| 200 | 248.08 | 262.53 | 301.41 | 334.38 |
| 250 | 246.77 | 261.35 | 300.45 | 333.52 |
| 300 | 244.79 | 259.99 | 299.56 | 332.66 |
| 350 | 243.76 | 258.74 | 298.54 | 331.75 |
| 400 | 242.13 | 257.46 | 297.52 | 330.85 |
| 450 | 240.46 | 256.21 | 296.49 | 329.91 |
| 500 | 238.78 | 254.86 | 295.44 | 328.96 |
| 550 | 237.06 | 253.49 | 294.39 | 328.01 |
| 600 | 235.33 | 252.12 | 293.33 | 327.06 |
| 650 | 233.56 | 250.74 | 292.27 | 326.11 |
| 700 | 231.73 | 249.32 | 291.21 | 325.16 |
| 750 | 229.83 | 247.88 | 290.14 | 324.2 |
| 800 | 227.83 | 246.41 | 289.06 | 323.23 |
| 850 | 225.15 | 244.78 | 288.04 | 322.29 |
| 900 | 224.05 | 243.81 | 286.96 | 321.33 |
| 950 | 220.94 | 242.03 | 285.87 | 320.36 |
| 1000 | 217.64 | 240.22 | 284.78 | 319.4 |

**Supplementary table 2.** The Debye temperature of ZnSe under B3 phase as a function of temperature at a given pressure. These data obtain from Figure 7(b).

| Temperature (K) | Debye temperature at a given Pressure (K) | | | |
| --- | --- | --- | --- | --- |
|  | 15 GPa | 20 GPa | 25 GPa | 30 GPa |
| 0 | 381.3 | 405.06 | 427.18 | 447.93 |
| 50 | 381.26 | 405.02 | 427.15 | 447.91 |
| 100 | 380.88 | 404.69 | 426.85 | 447.64 |
| 150 | 380.19 | 404.04 | 426.26 | 447.1 |
| 200 | 379.33 | 403.23 | 425.5 | 446.39 |
| 250 | 378.38 | 402.33 | 424.65 | 445.58 |
| 300 | 377.39 | 401.39 | 423.75 | 444.73 |
| 350 | 376.37 | 400.42 | 422.83 | 443.85 |
| 400 | 375.35 | 399.44 | 421.88 | 442.93 |
| 450 | 374.29 | 398.44 | 420.92 | 442.01 |
| 500 | 373.23 | 397.42 | 419.98 | 441.07 |
| 550 | 372.17 | 396.4 | 418.98 | 440.14 |
| 600 | 371.1 | 395.4 | 417.99 | 439.19 |
| 650 | 370.04 | 394.36 | 416.99 | 438.23 |
| 700 | 368.95 | 393.33 | 416 | 437.27 |
| 750 | 368.01 | 392.34 | 414.99 | 436.26 |
| 800 | 366.88 | 391.3 | 414.02 | 435.34 |
| 850 | 365.76 | 390.27 | 413.02 | 434.38 |
| 900 | 364.58 | 389.18 | 412.02 | 433.44 |
| 950 | 363.7 | 388.24 | 411.02 | 432.42 |
| 1000 | 362.6 | 387.2 | 410.04 | 431.47 |

**Supplementary table 3.** The Debye temperature of ZnSe under B1 phase as a function of temperature at a given pressure. These data obtain from Figure 7(c).

| Structure | B3 | | | | Structure | B1 | | | |
| --- | --- | --- | --- | --- | --- | --- | --- | --- | --- |
| Pressure (GPa) | *C_v_* at a given temperature (J·mol^-1^ K^-1^) | | | | Pressure (GPa) | *C_v_* at a given temperature (J·mol^-1^ K^-1^) | | | |
|  | 50 K | 300 K | 600 K | 1000 K |  | 50 K | 300 K | 600 K | 1000 K |
| 0.1 | 18.24 | 48.27 | 49.51 | 49.77 | 14.85 | 7.81 | 46.17 | 48.95 | 49.56 |
| 1 | 17.46 | 48.17 | 49.48 | 49.76 | 15 | 7.77 | 46.15 | 48.95 | 49.56 |
| 2 | 16.65 | 48.06 | 49.45 | 49.74 | 16 | 7.53 | 46.06 | 48.92 | 49.55 |
| 3 | 15.91 | 47.96 | 49.42 | 49.73 | 17 | 7.30 | 45.97 | 48.90 | 49.54 |
| 4 | 15.23 | 47.86 | 49.40 | 49.72 | 18 | 7.08 | 45.87 | 48.87 | 49.53 |
| 5 | 14.60 | 47.77 | 49.37 | 49.71 | 19 | 6.88 | 45.78 | 48.85 | 49.52 |
| 6 | 14.01 | 47.67 | 49.35 | 49.70 | 20 | 6.68 | 45.69 | 48.82 | 49.52 |
| 7 | 13.45 | 47.58 | 49.32 | 49.69 | 21 | 6.49 | 45.60 | 48.80 | 49.51 |
| 8 | 12.94 | 47.49 | 49.30 | 49.69 | 22 | 6.31 | 45.51 | 48.77 | 49.50 |
| 9 | 12.45 | 47.39 | 49.27 | 49.68 | 23 | 6.14 | 45.42 | 48.75 | 49.49 |
| 10 | 12.00 | 47.30 | 49.25 | 49.67 | 24 | 5.97 | 45.33 | 48.72 | 49.48 |
| 11 | 11.57 | 47.21 | 49.22 | 49.66 | 25 | 5.82 | 45.24 | 48.70 | 49.47 |
| 12 | 11.16 | 47.13 | 49.20 | 49.65 | 26 | 5.67 | 45.16 | 48.67 | 49.46 |
| 13 | 10.78 | 47.04 | 49.18 | 49.64 | 27 | 5.52 | 45.07 | 48.65 | 49.45 |
| 14 | 10.42 | 46.95 | 49.15 | 49.63 | 28 | 5.39 | 44.98 | 48.62 | 49.44 |
| 14.85 | 10.13 | 46.88 | 49.13 | 49.63 | 29 | 5.25 | 44.89 | 48.60 | 49.43 |
| - | - | - | - | - | 30 | 5.13 | 44.81 | 48.58 | 49.43 |

**Supplementary table 4.** The heat capacity of ZnSe under both phases as a function of pressure at a given temperature. These data obtain from Figure 8(a).

| Temperature (K) | *C_v_* at a given temperature (J·mol^-1^ K^-1^) | | | |
| --- | --- | --- | --- | --- |
|  | 0.1 GPa | 3 GPa | 9 GPa | 14 GPa |
| 0 | 0 | 0 | 0 | 0 |
| 50 | 18.24 | 16.65 | 12.94 | 10.42 |
| 100 | 37.17 | 36.00 | 32.80 | 30.11 |
| 150 | 43.62 | 42.96 | 41.06 | 39.35 |
| 200 | 46.25 | 45.84 | 44.65 | 43.56 |
| 250 | 47.54 | 47.26 | 46.46 | 45.72 |
| 300 | 48.27 | 48.06 | 47.49 | 46.95 |
| 350 | 48.70 | 48.55 | 48.12 | 47.72 |
| 400 | 48.98 | 48.87 | 48.53 | 48.22 |
| 450 | 49.18 | 49.09 | 48.82 | 48.57 |
| 500 | 49.32 | 49.24 | 49.03 | 48.82 |
| 550 | 49.43 | 49.36 | 49.18 | 49.01 |
| 600 | 49.51 | 49.45 | 49.30 | 49.15 |
| 650 | 49.57 | 49.52 | 49.39 | 49.26 |
| 700 | 49.61 | 49.57 | 49.46 | 49.35 |
| 750 | 49.65 | 49.62 | 49.52 | 49.42 |
| 800 | 49.69 | 49.65 | 49.56 | 49.48 |
| 850 | 49.71 | 49.68 | 49.60 | 49.53 |
| 900 | 49.73 | 49.70 | 49.63 | 49.57 |
| 950 | 49.75 | 49.73 | 49.66 | 49.60 |
| 1000 | 49.77 | 49.74 | 49.69 | 49.63 |

**Supplementary table 5.** The heat capacity of ZnSe under B3 phases as a function of temperature at a given pressure. These data obtain from Figure 8(b).

| Temperature (K) | *C_v_* at a given temperature (J·mol^-1^ K^-1^) | | | |
| --- | --- | --- | --- | --- |
|  | 15 GPa | 20 GPa | 25 GPa | 30 GPa |
| 0 | 0 | 0 | 0 | 0 |
| 50 | 7.77 | 6.68 | 5.82 | 5.13 |
| 100 | 26.54 | 24.75 | 23.15 | 21.71 |
| 150 | 36.92 | 35.61 | 34.38 | 33.22 |
| 200 | 41.95 | 41.05 | 40.20 | 39.37 |
| 250 | 44.61 | 43.98 | 43.37 | 42.78 |
| 300 | 46.15 | 45.69 | 45.24 | 44.81 |
| 350 | 47.12 | 46.77 | 46.43 | 46.10 |
| 400 | 47.76 | 47.49 | 47.22 | 46.96 |
| 450 | 48.20 | 47.99 | 47.77 | 47.56 |
| 500 | 48.52 | 48.35 | 48.17 | 48.00 |
| 550 | 48.76 | 48.62 | 48.47 | 48.33 |
| 600 | 48.95 | 48.82 | 48.70 | 48.58 |
| 650 | 49.09 | 48.98 | 48.88 | 48.77 |
| 700 | 49.20 | 49.11 | 49.02 | 48.93 |
| 750 | 49.29 | 49.21 | 49.13 | 49.05 |
| 800 | 49.37 | 49.30 | 49.23 | 49.16 |
| 850 | 49.43 | 49.37 | 49.30 | 49.24 |
| 900 | 49.48 | 49.42 | 49.37 | 49.31 |
| 950 | 49.52 | 49.47 | 49.42 | 49.37 |
| 1000 | 49.56 | 49.48 | 49.47 | 49.43 |

**Supplementary table 6.** The heat capacity of ZnSe under B1 phases as a function of temperature at a given pressure. These data obtain from Figure 8(c).

**Author contributions**

Acquisition of data: T. Y., X. Z., J. J., J. W. Drafting of the table: T. Y. and X. Z. Critical revision of the table: J. J. and J. W.
